# A Pilot Study of ^18^F-DCFPyL PET/CT or PET/MRI and Ultrasound Fusion Targeted Prostate Biopsy for Intra-Prostatic PET-Positive Lesions

**DOI:** 10.3389/fonc.2021.612157

**Published:** 2021-03-05

**Authors:** Yachao Liu, Hongkai Yu, Jiajin Liu, Xiaojun Zhang, Mu Lin, Holger Schmidt, Jiangping Gao, Baixuan Xu

**Affiliations:** ^1^Department of Nuclear Medicine, Chinese People’s Liberation Army General Hospital, Beijing, China; ^2^Department of Urology Surgery, Chinese People’s Liberation Army General Hospital, Beijing, China; ^3^Magnetic Resonance Collaboration, Diagnostic Imaging, Siemens Healthineers Ltd., Shanghai, China; ^4^Magnetic Resonance Education, Customer Services, Siemens Healthcare GmbH, Erlangen, Germany

**Keywords:** ^18^F-DCFPyL, PET/CT, PET/MR, biopsy, prostate cancer

## Abstract

**Objectives:**

The purpose of this study was to evaluate the feasibility and diagnostic performance of prostate-specific membrane antigen (PSMA) based ^18^F-DCFPyL PET/CT-ultrasound (PET/CT-US) or PET/MRI-ultrasound (PET/MRI-US) fusion targeted biopsy for intra-prostatic PET-positive lesions.

**Methods:**

From April 2018 to November 2019, we prospectively enrolled 55 candidates to perform PET/CT-US or PET/MRI-US fusion targeted biopsies for solitary PET-positive prostate lesions (two to four cores/lesion). The positive rates of prostate cancer based on patients and biopsy cores were calculated respectively. With reference to the pathological results of biopsy cores, the MR signal characteristics in the area of the PET-positive lesion were analyzed for the patients who underwent PET/MRI.

**Results:**

A total of 178 biopsy cores were taken on the 55 patients. One hundred forty-six biopsy cores (82.0%, 146/178) from 51 (92.7%, 51/55) patients were positive for prostate cancer; 47 (85.5%, 47/55) were clinically significant prostate cancer. It is noteworthy that nine patients underwent both ^18^F-DCFPyL PET/CT and PET/MRI examinations; the seven patients with prostate cancer showed abnormal MR signal in the area of the PET-positive lesion while the other two patients with prostatic hyperplasia and prostatitis showed normal MR signal in the area of the PET-positive lesion.

**Conclusion:**

This study indicated that ^18^F-DCFPyL PET/CT-US or PET/MRI-US fusion targeted prostate biopsies may be valuable for prostate cancer diagnosis and have a high detection rate of clinically significant prostate cancer for PET-positive lesions. PET/MR can rule out some false PET-positive lesions, which may potentially reduce unnecessary prostate biopsies.

## Introduction

Prostate cancer remains one of the most common male malignancies worldwide. Systematic 12-core transrectal ultrasound-guided prostate biopsy and histopathology are the most commonly used techniques for the diagnosis of prostate cancer before radical prostatectomy (1). However, biopsies are invasive, painful, and prone to potential complications. Normal prostate tissue, benign prostate diseases, and clinically insignificant prostate cancer are often detected by this conventional biopsy scheme. In addition, this conventional approach is poor at sampling the anterior, midline, and apex of the prostate, which leads to the underdiagnosis of patients with clinically significant prostate cancer.

Much progress has been made in recent years towards developing a targeted prostate biopsy. Clinicians are constantly exploring new methods, such as direct in-bore MRI guidance and image fusion guidance targeted prostate biopsy, to improve the detection of clinically significant prostate cancer and reduce the number of biopsy procedures and associated complications (2). However, 24% of men with negative multiparametric MRI have a significant risk of harboring clinically significant prostate cancer (3).

PSMA is a type II transmembrane glycoprotein with enzymatic carboxypeptidase activity. PSMA is overexpressed on almost all types of prostate cancer cells, making PMSA an ideal target for the diagnosis and treatment of prostate cancer. Compared with conventional imaging modalities, such as CT and MR, both ^68^Ga and ^18^F labeled PSMA PET imaging has a higher sensitivity and specificity for prostate cancer (4–7). ^18^F-DCFPyL is a very promising ^18^F-labeled PSMA tracer that is currently under investigation. A previous study showed that ^18^F labeled PSMA provides better image quality and the ability to display small lesions (8). The purpose of our study was to explore the feasibility and diagnostic performance of ^18^F-DCFPyL PET/CT or PET/MRI and ultrasound (PET/CT-US or PET/MRI-US) fusion-targeted prostate biopsy for intra-prostatic PET-positive lesion diagnosis.

## Materials and Methods

### Study Population

Between April 2018 and November 2019, 213 consecutive patients performed ^18^F-DCFPyL PET/CT or PET/MRI because of elevated PSA, digital rectal examination, ultrasound, or MRI suspected prostate cancer. The patients with solitary PET-positive prostate lesions were assessed for eligibility for ^18^F-DCFPyL PET targeted biopsy and informed of the potential harms and benefits of this method. The inclusion criteria were: (1) ^18^F-DCFPyL PET/CT or PET/MRI showed solitary radioactive concentration (PET-positive lesion) in the prostate; and, (2) the solitary PET-positive lesion involved less than one half of one lobe; and, (3) a targeted biopsy performed by ^18^F-DCFPyL PET/CT-US or PET/MRI-US targeted at the solitary intraprostatic PET-positive lesion. Patients were excluded if (1)^18^F-DCFPyL PET/CT or PET/MRI showed multiple or no PET-positive lesion in the prostate; and (2) they chose systematic biopsy instead of our targeted biopsy way or refused any prostate biopsy. All procedures were approved by the local ethics board and all the enrolled subjects provided informed consent.

### 18F-DCFPyL PET/CT and PET/MRI Examinations

^18^F-DCFPyL was synthesized by our nuclear medicine department (radiochemical purity > 98%, specific activity 54–90 GBq/μmol). Quality control report was provided in **Table 1**.

**Table 1:** Quality control report.

Test	Specification	Average Original (n = 4)
Initial appearance	Clear, colorless solution, no visible particulate matter	Conforms
Appearance, 240 min after end of synthesis	Clear, colorless solution, no visible particulate matter	Conforms
Initial radiochemical purity, %	≥95%	100%
pH, initial	4.5–8.5	6.5
Chemical purity	DCFPyL	3.87 ± 0.13 μg/ml
Yield	≥20 mCi [18F]DCFPyL (referenced to assay recorded at end of filtration)	347 ± 45 mCi
Specific activity	≥1,000 mCi/μmol of [18F]DCFPyL (referenced to end of filtration)	65 ± 23 GBq/μmol
Residual solvent analysis	Acetonitrile ≤400 ppm Tetrabutylammonium ≤400 ppm	0 ppm 0 ppm
Radionuclidic identity	t1/2 = 105–115 min	109.8 ± 2.3 min
Radionuclidic purity	99.5% associated with ^18^F (0.511 and 1.022 MeV)	Conforms
Identity (high-performance liquid chromatography, HPLC)	HPLC retention time matches reference standard	Conforms
Filter integrity	Bubble point ≥13 psi	17.3 ± 0.6 psi
Endotoxin	≤15 EU/ml	<5 EU/ml
Sterility	No growth observed	Conforms

PET/CT was performed from the ears to the upper thigh on a Siemens Biograph 64 operating in 3D emission mode with CT-derived attenuation correction (120 kV, 100 mAs, 5.0 mm Slice, 0.9 Pitch). The PET acquisition time was 2 min per bed position. CT maps were used for PET attenuation correction. PET data were reconstructed using ordered subset expectation maximization (OSEM; 3 iterations, 21 subsets, 168 × 168 matrix) and a transaxial resolution of 5.0 mm (full-width at half-maximum).

PET/MRI was performed on a hybrid PET/MRI scanner (Biograph mMR, Siemens Healthcare, Erlangen). The MR protocol consisted of T1W fast spin echo (2D, transversal, TR 500 ms, TE 13 ms, flip angle 150°, 15 slices, Slice thickness 5 mm, field of view (FOV) 300 × 300, voxel size 1.2 × 1.2 × 5.0 mm^3^), T2W (transversal, TR 3810 ms, TE 78 ms, flip angle 150°, 20 slices, Slice thickness 3 mm, FOV 240 × 240, voxel size 0.8 × 0.8 × 3.0 mm^3^) fast spin echo, high b value DWI (2D, transversal, TR 6500 ms, TE 93 ms, 20 slices, Slice thickness 3 mm, FOV 380 × 380, voxel size 2.4 × 2.4 × 3.0 mm^3^, b-values 0, 1,000 and 2,000 s/mm^2^), and PET acquisition for the pelvic region. PET images were reconstructed with 3 iterations and 21 subsets. MRI‐based attenuation correction was applied using DIXON‐volume interpolated breath-hold examination (VIBE) sequences comprising in‐ and opposed‐phase as well as fat‐ and water‐saturated images.

### Prostate Biopsy Procedure

**Figure 1:**
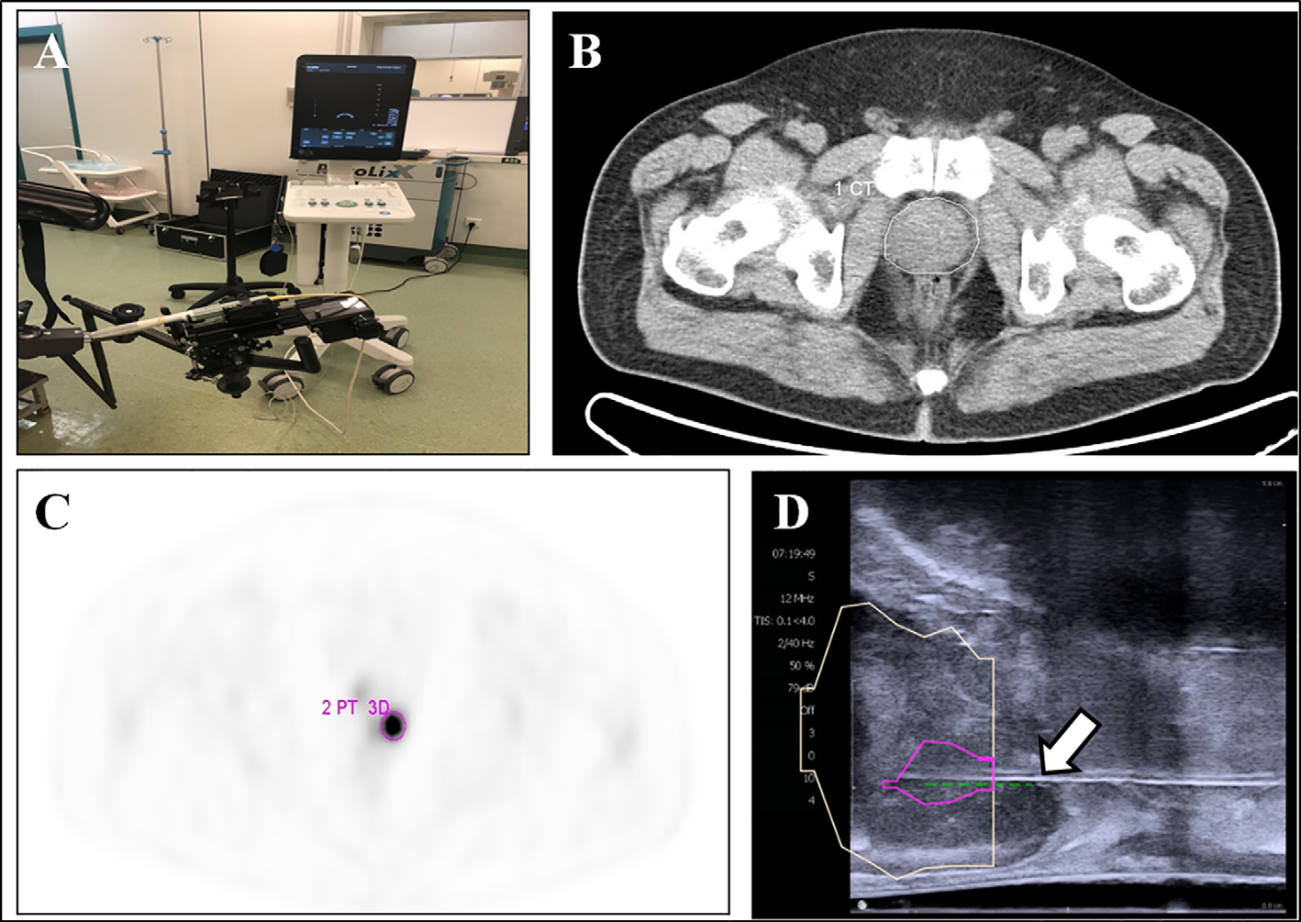
^18^F-DCFPyL PET/CT-US or PET/MRI-US fusion targeted prostate biopsy for the intraprostatic PET-positive lesions were performed with the BK Predictive Fusion prostate biopsy system **(A)**. The boundaries of the prostate were delineated on the CT image (**B**, white circle). The PET-positive lesion was marked as the target for biopsy (**C**, pink circle). The previous delineated prostate and PET-positive lesion from PET/CT was registered to the prostate volume acquired from the three-dimensional transrectal ultrasonography; the puncture needle (**D**, arrow) then reached the target biopsy area.

^18^F-DCFPyL PET/CT-US or PET/MRI-US fusion targeted prostate biopsy for the intra-prostatic PET-positive lesions were performed with the BK Predictive Fusion prostate biopsy system (BK Medical Technology Shanghai Co., Ltd). Patients were given fluoroquinolone antibiotic prophylaxis and prescribed enemas on the day before the procedure, and again 3 h before the procedure. Before the targeted biopsy, the ^18^F-DCFPyL PET/CT or PET/MRI imaging data were imported into the BK Predictive Fusion prostate biopsy system workstation, and the boundaries of the prostate were delineated on CT or T2W images. A standardized uptake value (SUV) of 2.5 was used to delineate the boundary of PET-positive lesions marked as targets for biopsy. The SUVmax, SUVmean, and volume of lesions were also recorded for further analysis. The patients were placed in the Trendelenburg position and administered local anesthesia of 1% lidocaine. During the biopsy procedure, the previous delineated prostate volume from the CT or T2W images was then registered with the prostate volume acquired from the three-dimensional transrectal ultrasonography with real-time tracking of the ultrasound probe. Subsequently, an algorithm determined the precise three-dimensional real-time information about the localization of targets in the PET-positive lesion for needle placement during the ultrasound-guided biopsy. Transrectal biopsies were performed with two to four cores for each PET-positive lesion. A schematic diagram of a targeted prostate biopsy procedure is shown in **Figure 1**.

Compared with targeted biopsy, systematic biopsy causes greater suffering to patients, both psychologically and physically (post-biopsy complications). Some patients, particularly elders, are unwilling to undergo systematic biopsy. In contrast, ^18^F-DCFPyL PET positive lesions have high diagnostic value for prostate cancer. For patients with single PET positive lesion, targeted biopsy (two to four cores) alone can achieve good detection rate and reduce patients’ pain. However, only targeted biopsy for PET-positive lesion might miss some PET-negative prostate cancer lesions. The advantages and disadvantages of the two biopsy methods were informed to the patients.

### Histopathologic Analysis

The prostate biopsies were analyzed by two pathologists, each with over 10 years of experience in prostate pathology. Analyzed biopsy features include the total number of biopsy cores, percentage of cores involving adenocarcinoma, and the number of positive biopsy cores. In our study, clinically significant prostate cancer was defined as the presence of a single biopsy core indicating disease of Gleason score 3 + 4 (Gleason sum of 7) or greater (the Gleason score is composed of a primary [most predominant] grade plus a secondary [highest non-predominant] grade); the range for a primary or secondary grade is from 3 to 5, with the Gleason sum ranging from 6 to 10 (3, 9–12).

## Results

**Figure 2:**
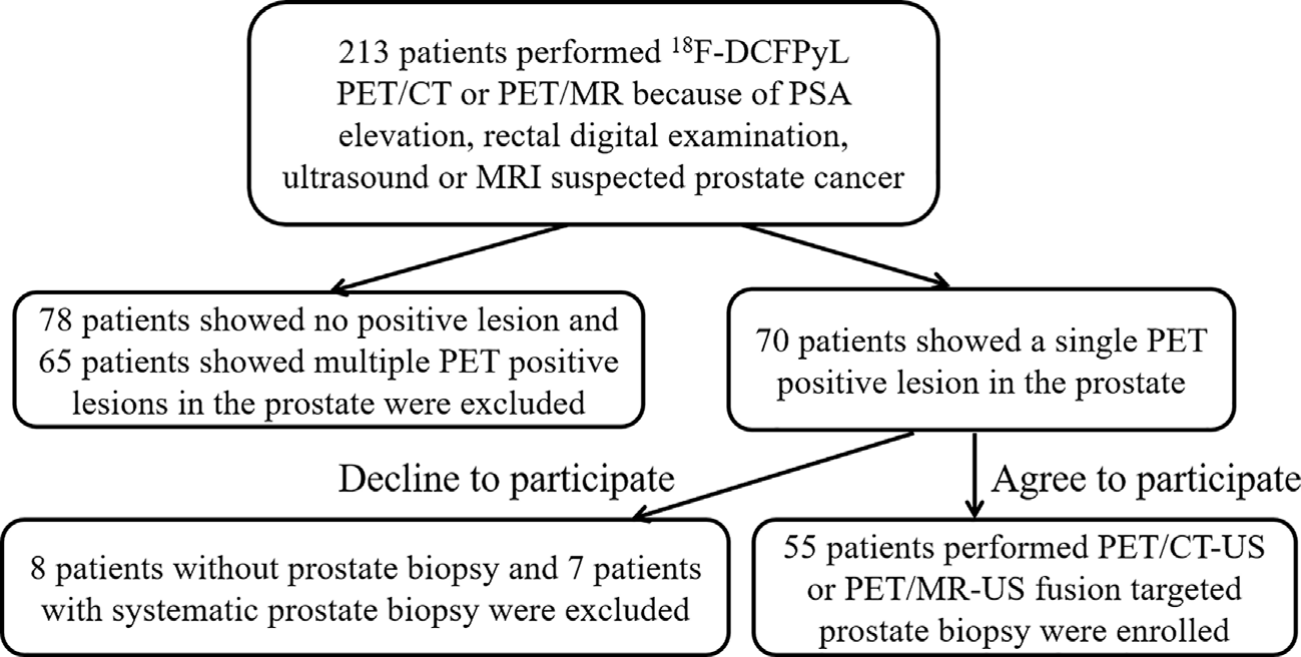
Prostate biopsy algorithm of study subjects.

A total of 55 patients (mean age: 67, range: 49–84) with solitary PET-positive prostate lesions agreed to undergo ^18^F-DCFPyL PET/CT-US or PET/MRI-US fusion targeted prostate biopsy were enrolled in this study (**Figure 2**). Among them, two cases had previous negative prostate biopsy and 53 cases were biopsy-naive. Finally, no significant complications occurred in any patient after the biopsy. An overview of the study population is shown in **Table 2**.

**Table 2:** Clinical characteristics of the 55 patients enrolled in the study.

Patient characteristics	Total(n = 55)	PET/CT-US(n = 32)	PET/MRI-US(n = 23)
Age (year; mean ± SD)	67.02 ± 9.05	67.91 ± 9.43	65.78 ± 8.53
PSA (ng/ml; mean ± SD)	14.37 ± 10.31	13.82 ± 7.97	15.13 ± 13.05
Target volume (cm^3^; mean ± SD)	3.68 ± 2.18	4.14 ± 2.17	3.04 ± 2.08
SUVmax (mean ± SD)	15.47 ± 12.25	15.19 ± 12.53	15.85 ± 12.10
SUVmean (mean ± SD)	7.07 ± 4.68	7.37 ± 5.22	6.65 ± 3.87
Total number of cores from targeted biopsy	178	103	75
Positive cores on targeted biopsy (%)	146 (82.0%)	85 (82.5%)	61 (81.3%)
Men with prostate cancer	51 (92.7%)	30 (93.8%)	21 (91.3%)
No. Gleason score			
Gleason 3 + 3	4	2	2
Gleason 3 + 4	13	5	8
Gleason 4 + 3	16	10	6
Gleason 4 + 4	10	5	5
Gleason 5 + 3	1	1	0
Gleason 4 + 5	7	7	0
Men with clinically significant prostate cancer (%)	85.5% (47/55)	87.5% (28/32)	82.6% (19/23)

Among the 55 patients, 32 patients received PET/CT examinations along, 14 patients received PET/MRI examinations along, and 9 patients underwent both PET/CT and PET/MRI scans sequentially within 2 h. The average volume of the target biopsy area was 3.68 ± 2.18 cm^3^ (range: 0.7–9.82 cm^3^). The average SUVmax ± SD was 15.47 ± 12.25 (range: 4.36–59.34), and the average SUVmean ± SD was 7.07 ± 4.68 (range: 3.39–30.30).

A total of 178 core biopsies were performed, 146 (82.0%, 146/178) samples were malignant. According to the biopsy pathology, fusion-targeted biopsy identified 51 (92.7%, 51/55) patients as having prostate cancer; 47 (85.5%, 47/55) of these 55 patients were clinically significant prostate cancer while 4 (7.3%, 4/55) were clinically insignificant prostate cancer. The detection rates of clinically significant prostate cancer by PET/CT-US and PET/MRI-US were 87.5% (28/32) and 82.6% (19/23), respectively.

Among the 55 patients, 32 were examined using only ^18^F-DCFPyL PET/CT; 28 were diagnosed with clinically significant cancer, 2 with clinically insignificant cancer, and 2 with benign prostatic hyperplasia.

Of the 14 patients examined using only ^18^F-DCFPyL PET/MR, 13 were diagnosed with clinically significant cancer, and 1 was diagnosed with clinically insignificant cancer. All of these 14 patients showed abnormal MR signals (low T2 signal, high DWI signal, decreased ADC value) in the area of PET-positive lesions.

**Figure 3:**
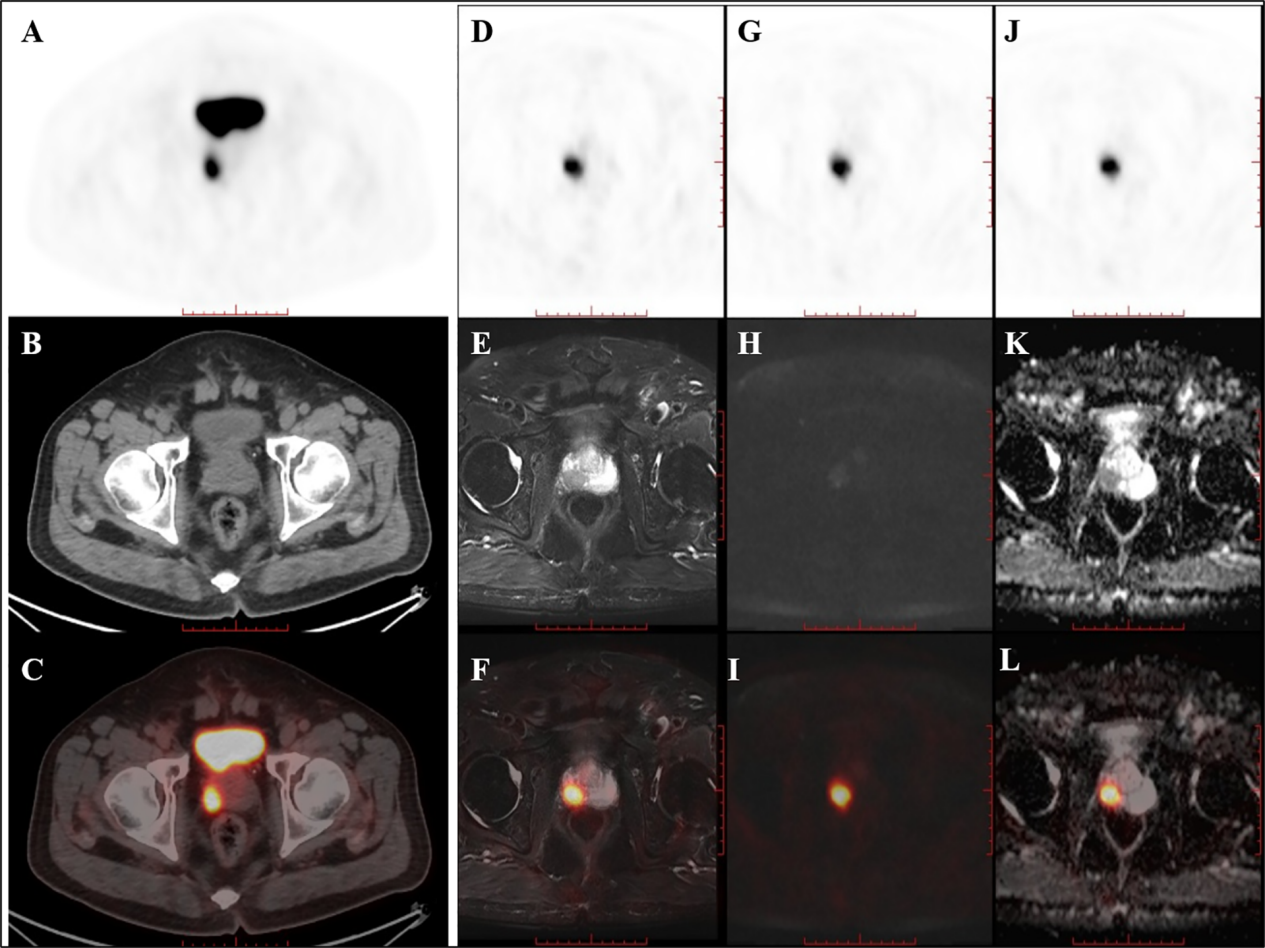
PET/CT found one PET-positive lesion in the prostate gland (**A:** PET, **B**: CT, **C**: fused PET/CT). PET/MRI showed short T2 signal (**D**: PET, **E**: T2WI, **F**: fused PET/T2WI), high DWI signal (**G**: PET, **H**: DWI, **I**: fused PET/DWI), and a decreased ADC value at the site of the PET-positive lesion (**J**: PET, **K**: ADC map, **L**: fused PET/ADC map). The subsequent pathology confirmed prostate cancer.

**Figure 4:**
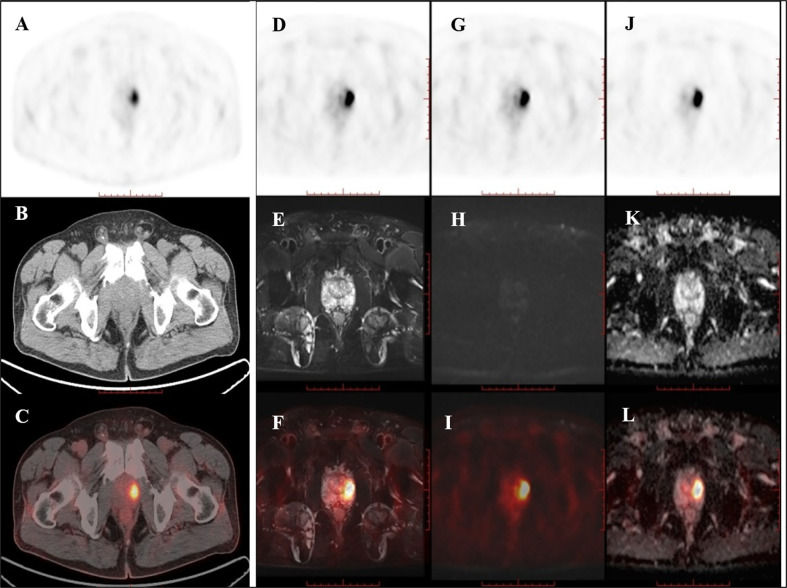
PET/CT found one PET-positive lesion in the prostate gland (**A**: PET, **B**: CT, **C**: fused PET/CT). PET/MRI showed no obviously abnormal signal on T2WI (**D**: PET, **E**: T2WI, **F**: fused PET/T2WI), DWI (**G**: PET, **H**: DWI, **I**: fused PET/DWI), or the ADC map at the site of the PET-positive lesion (**J**: PET, **K**: ADC map, **L**: fused PET/ADC map). The subsequent pathology confirmed prostatic hyperplasia and prostatitis.

Of the nine patients that underwent successive examinations of ^18^F-DCFPyL PET/CT and PET/MRI, seven examinations showed abnormal MR signal (decreased T2 signal, low ADC value, and increased DWI signal at both 1,000 and 2,000 s/mm^2^) in the area of the PET-positive lesions (a typical case is shown in **Figure 3**), and the subsequent pathology confirmed prostate cancer in all cases. The other two patients showed normal MR signal in the area of the PET-positive lesions (a typical case is shown in **Figure 4**), and the subsequent biopsy confirmed prostatic hyperplasia and prostatitis.

## Discussion

This preliminary study demonstrates that for patients with clinical suspicion of prostate cancer and PSMA (^18^F-DCFPyL) PET-positive lesions, using PET/CT-US or PET/MRI-US fusion-targeted prostate biopsy had a high detection rate of clinically significant prostate cancer. Furthermore, PET/MRI was able to identify the false positive lesions by PSMA PET. To our knowledge, this is the first study to explore the feasibility and diagnostic performance of PSMA PET/CT-US and PET/MRI-US fusion targeted prostate biopsy.

The trade-off between detection rate and the number of biopsy cores is a major concern with men suspected of prostate cancer. As the number of biopsy cores increases, the detection rate of prostate cancer increases but the portion of low-grade prostate cancer detected also increases. In addition, more biopsies lead to post-biopsy complications, including urinary retention, infection, hematuria, and hematochezia. Although prostate cancer screening strategies with repeated PSA testing and an extended-core prostate biopsy protocol reduces the incidence and mortality of advanced disease, it also leads to a significant proportion of overdiagnosis and consequently overtreatment for some low-risk tumors that may not result in symptoms or death from the disease during a patient’s lifetime (13). Overdiagnosis and overtreatment of non-lethal tumors expose patients with insignificant prostate cancer to unnecessary side-effects. Thus, it is critical that the location and characteristics of prostate cancer are known before making treatment decisions.

A systematic 12-core transrectal ultrasound-guided biopsy is clinically recommended (14), and the rate of prostate cancer detection for a first systematic transrectal ultrasound-guided biopsy is typically 30–50% (15). Even the extended biopsy schemes with more than 12 cores may still miss almost a third of prostate cancers (16). Magnetic resonance imaging and ultrasonography (MRI-US) fusion targeted prostate biopsy has the advantages of accurate localization of lesions and real-time imaging, and it has been gradually applied to clinical practice. According to one systematic review, the median detection rate of prostate cancer was 43.4% with the standard biopsy strategy *versus* 50.5% with MRI-US image fusion targeted biopsy (17). No obvious advantage of MRI/US fusion-guided biopsy was observed in terms of the cancer detection rate compared to a standard systematic biopsy (18).

The accurate detection and delineation of intra-prostatic tumors are important for diagnosis and treatment decisions for patients with primary prostate cancer. Radionuclide ^18^F or ^68^Ga labeled PSMA PET imaging has great value in the diagnosis of prostate cancer. After prostatectomy, a histology map of the prostate was reconstructed, and the histological extension of each segment (132 segments from six patients) of the prostate was compared with PSMA PET images, the correlation of histological results with PSMA PET images showed a specificity and sensitivity of 92%, respectively (19). Some studies have validated the performance of PSMA PET/CT to define the gross tumor volume (GTV) through comparison with histology and have reported good results with high sensitivity and specificity in the detection of primary prostate cancer (19–21). In a recent study, 31 patients with previously negative prostate biopsy, but persistently elevated serum PSA, were imaged with ^68^Ga-labeled PSMA PET/CT and then underwent both standard systematic biopsy and PET/CT-US fusion targeted biopsy (9): Among the 13 patients who were negative on PSMA PET imaging, none were diagnosed with clinically significant cancer; in the 18 patients positive by PSMA PET imaging, PET/CT-US fusion targeted biopsy detected all 12 patients with clinically significant cancer while standard systematic biopsy detected only 10 patients. These preliminary results suggest that PSMA PET might be a useful tool to identify and define malignant lesions prior to prostate biopsy. The results of our study showed that fusion of ^18^F-DCFPyL PET/CT or PET/MRI with ultrasound is beneficial and feasible for guiding targeted prostate biopsy. In addition, this preliminary result indicates that ^18^F-DCFPyL PET/CT-US or PET/MRI-US fusion targeted prostate biopsy may be a good way to reduce over-diagnosis of clinically insignificant prostate cancer and improve detection of clinically significant cancer. This method allows urologists to progress from blind, systematic biopsies to biopsies that are mapped, targeted, and tracked. More rigorous and comprehensive studies should be designed to prove the clinical value of PET/CT-US and PET/MRI-US fusion targeted prostate biopsies.

In this study, there were only four patients whose biopsy pathology was negative for prostate cancer yielding a 7.3% (4/55) false-positive rate for the ^18^F-DCFPyL PET-positive lesions. There are several possibilities why the PET-positive lesions were not malignant. According to a case report, a patient with 2two focal PSMA-positive areas in the prostate gland, one corresponded to prostate cancer (Gleason score 4 + 3), while the other had no evidence of malignancy despite high PSMA expression on immunohistochemistry (22). Another explanation for the false positive cases may be the motion during PET/CT examinations. Even if a true prostate cancer lesion is correctly declared by ^18^F-DCFPyL PET, there is a possibility that it may be missed on biopsy if PET and CT indicated different locations. In contrast, urologists can consider the lesion localization provided by MR images when performing PET/MRI-US fusion targeted prostate biopsies to help improve the target position precision of biopsy cores.

Prostate cancer is typically characterized by abnormal MR signal (low signal on T2-weighted images, high signal on DWI, and low ADC value) and high PSMA uptake on PET/MR. Benign prostate diseases such as prostatitis, benign prostatic hyperplasia (BPH), and scarring are heterogeneous and may sometimes appear similar to prostate cancer on MRI (23), but they generally do not show obvious PSMA uptake. Prostate tumors with small size and low grade can have atypical manifestations on PSMA PET/MR. When compared with the radical prostatectomy specimens pathology, 5.9% intra-prostatic tumors were non-avid for ^68^Ga-PSMA PET, and 5.4% intra-prostatic tumors were not detected by mp-MRI (24). PET/MRI is a new multi-modal imaging technique that is expected to improve the diagnostic performance of imaging, especially in cases where soft-tissue evaluation is crucial, such as prostate cancer (4, 25). In our study, nine patients underwent both ^18^F-DCFPyL PET/CT and PET/MRI successively, and seven of these patients had abnormal MR signal in the area of the PET-positive lesions that were prostate cancer. The other two cases showed no obvious abnormal MR signal in the area of the PET-positive lesions were hyperplasia and prostatitis. PET/MRI is expected to further improve the prostate biopsy efficacy by reducing unnecessary prostate biopsies in some patients with PET-positive and MR-negative lesions.

Theoretically, targeted biopsies only for PSMA PET-positive lesion cannot rule out the presence of prostate cancer lesion in the PET-negative area of prostate. Thus, systematic prostate biopsy can provide added value to PET targeted biopsy. Zhang LL et al. (26) performed targeted biopsies alone for 25 patients with PSMA-avid lesions, and 21 patients were diagnosed with prostate cancer by targeted biopsy. The other four patients with initially negative by targeted biopsy underwent supplementary systematic biopsy, two of them were still negative, and two patients were confirmed as prostate cancer by the supplementary systematic biopsy. To our knowledge, there is no published literature making direct comparison between systematic biopsy and targeted biopsy in the same patient cohort.

PSMA labeled ligands appear very promising for diagnosis and treatment of prostate cancer (27). While MRI has been effective in the detection of significant prostate cancer, its use in the identification and quantification of extra-prostatic disease is limited. This gap is now being filled by PSMA PET (28). Published studies (4, 29) have shown that PSMA PET (PET/CT or PET/MRI) exceeds MRI in the diagnosis and characterization of prostate cancer. A systematic review and meta-analysis from 13 studies showed the overall pooled sensitivity of PSMA PET/CT for staging in prostate cancer were 92% (30). MRI-US fusion-guided biopsies detected more clinically significant cancers than standard biopsy techniques (12, 17, 31–33). In our study, prostate biopsies performed on targeted ^18^F-DCFPyL PET-positive lesions of 55 patients had a high detection rate (51/55, 92.7%) of prostate cancer, and a high proportion (85.5%, 47/55) were clinically significant prostate cancer.

There are several limitations to our study. Firstly, the sample size was small, and the subsets of those getting PET/CT-US and PET/MRI-US guided biopsy were not randomized and prospectively powered but were rather convenience sample. Considering the limited number of cases in this study, we were unable to compare the diagnostic value between PET/CT-US and PET/MRI-US. Secondly, this study only used ^18^F-DCFPyL PET/CT-US and PET/MRI-US guided biopsy for targeted PET-positive lesions; therefore, we were unable to directly compare them with the systematic prostate biopsies. Thirdly, this study used the biopsy pathology for the diagnosis of prostate cancer and did not compare the pathological results of the biopsy with radical prostatectomy. This is because some patients included in this study were given endocrine therapy before the surgical operation, which will lead to the failure of the postoperative Gleason score evaluation, and other patients did not undergo radical prostatectomy due to advanced age or other factors. Lastly, although PSMA PET/CT-US or PET/MRI-US fusion targeted biopsy is of high diagnostic value, it is costly and can only be available in some general hospitals with the ability to synthesize PSMA labeled ligands and the equipment of PET/CT or PET/MRI scanner.

## Conclusion

In this study, ^18^F-DCFPyL PET/CT-US or PET/MRI-US fusion-targeted prostate biopsy proved to be feasible for prostate cancer diagnosis due to its high detection rate of clinically significant prostate cancer. PET/MR can rule out some false PET-positive lesions, which may potentially reduce unnecessary prostate biopsies. For patients with pacemakers or claustrophobia, ^18^F-DCFPyL PET/CT-US guided prostate biopsy remains a good alternative.

## Data Availability Statement

The original contributions presented in the study are included in the article/supplementary material. Further inquiries can be directed to the corresponding authors.

## Ethics Statement

The studies involving human participants were reviewed and approved by the Ethics Committee of Chinese PLA General Hospital. The patients/participants provided their written informed consent to participate in this study.

## Author Contributions

BX and JG conceived of the presented idea. YL and HY developed the theory and performed the computations. JL, XZ, HS, and ML verified the analytical methods. All authors provided critical feedback and helped shape the research, analysis, and manuscript and discussed the results. All authors contributed to the article and approved the submitted version.

## Funding

This work was financially supported by the National Natural Science Foundation of China project (No. 81571715), Achievement Transformation project of Chinese PLA General Hospital (No. 2018-TM-07), and Special scientific research topic of health care of Chinese PLA General Hospital (No. 19BJZ19).

## Conflict of Interest

Author ML was employed by company Siemens Healthineers Ltd., China. Author HS was employed by company Siemens Healthcare GmbH, Germany.

The remaining authors declare that the research was conducted in the absence of any commercial or financial relationships that could be construed as a potential conflict of interest.
